# Fast-Growing Meningioma in a Woman Undergoing Fertility Treatments

**DOI:** 10.1155/2016/3287381

**Published:** 2016-12-26

**Authors:** Adam Patterson, Abdurrahim Elashaal

**Affiliations:** ^1^Schulich School of Medicine & Dentistry, Windsor, ON, Canada; ^2^Department of Neurosurgery, Windsor Regional Hospital, Windsor, ON, Canada

## Abstract

Meningiomas have long been known to be associated with sexual hormones. We discuss here the case of a woman with a huge meningioma that rapidly grew over the course of a couple years while the patient was simultaneously taking fertility treatments. There is substantial evidence suggesting that fertility treatments can fuel the growth of meningiomas. The potential risks should be considered in women with a previous or family history of meningiomas who plan to undergo fertility treatment. These patients need to be evaluated and a screening imaging of brain MRI (Magnetic Resonant Imaging) should be offered in the middle or toward the end of such a treatment to control and prevent complications of these meningiomas.

## 1. Introduction

Meningiomas are the most commonly diagnosed intracranial neoplasm [1]. Many hypothesize that there is a link between meningiomas and female sex hormones. There is a female to male preponderance of two to one for meningiomas, and meningiomas are more common during puberty and menopause [1]. Previous cytological analysis of meningiomas showed that two-thirds of meningiomas contain progesterone receptors, and 10 percent of meningiomas contain estrogen receptors. There is a positive relationship between meningioma growth and with BMI [2]. The precise relation of females sex hormones with meningioma is unclear at this point. Most meningiomas display very slow growth: incidental meningiomas have an average annual growth rate of approximately 0.8 cm^3^ or a doubling time of approximately 22 years [3, 4]. There have been similar cases in the literature regarding meningiomas that were unusually large and fast-growing in various hyperestrogenic and hyperprogestogenic states. We report on the case of a remarkably large and fast-growing meningioma in a patient undergoing fertility treatments.

## 2. Case Report

**Figure 1:**
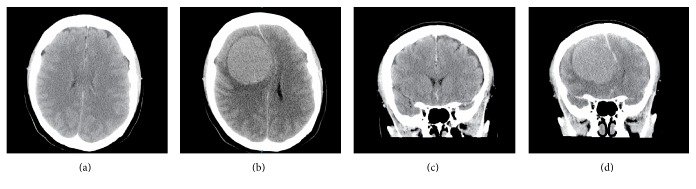
CT scan of the patient, displaying the rapid rate of growth of the meningioma: (a) axial view December 2012; (b) axial view December 2014; (c) coronal view 2012; (d) coronal view 2014.

**Figure 2:**
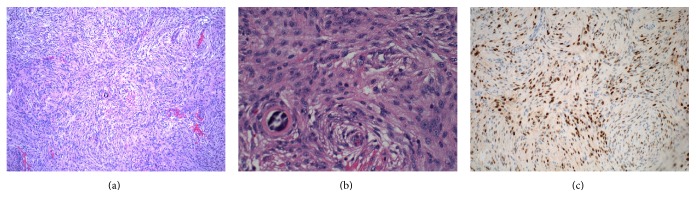
Histology of the tumor: (a) low magnification (×100) showing typical features of meningioma; (b) high magnification (×400) showing some evidence of nuclear atypia; (c) immunohistochemical staining showing the tumor to be progesterone receptor positive (×200 magnification).

A 36-year-old right-handed female presented with a two-week history of left leg weakness and altered level of consciousness, with a background two-year history of increasingly severe headaches and back pain. After taking CT imaging of the brain, a 6.3 × 5.7 × 6 cm mass was found in the right frontal lobe (Figures 1(b) and 1(d)). Previous CT without contrast performed two years previously (December 2012) because of increasingly severe headaches did not show a mass, though in retrospect it did show a focal area of density where the meningioma would later present (see Figures 1(a), and 1(c)). The patient was informed of the mass and consented to have surgery. A craniotomy was performed, and the tumor was excised without any complications. After surgery the patient did well, and her symptoms resolved. There were no further neurological symptoms or findings since then, and the patient remained tumor-free at her one-year follow-up. Histological examination of the specimen was consistent with grade II meningioma with atypia (Figures 2(a) and 2(b)). Progesterone staining was positive (Figure 2(c)).

The patient had undergone fertility treatment in November 2013, which involved a month long course of clomiphene citrate and progesterone as well as chorionic gonadotropins. This was unsuccessful at producing a viable gestation. The patient also had a previous fertility treatment in 2006 from July to September. This consisted of follitropin, progesterone, nafarelin acetate, and chorionic gonadotropins and was successful.

## 3. Discussion

The case we are reporting, a rapid growth of a meningioma after increased exposure to female sex steroids, draws many parallels with other causes within the literature. One case, for instance, reported on a 37-year-old right-handed female, with a prior history of three cycles of fertility treatment, who presented with a massive parasagittal meningioma [5]. A 29-year-old female presented with acute hemorrhage of a rhabdoid meningioma two weeks after initiating treatments of clomiphene citrate [6]. Furthermore, there have been multiple cases of meningiomas with accelerated growth during pregnancy [7, 8].

This case further substantiates the suggested relationship between meningiomas and female sex hormones. Firstly, the size of the tumor was significantly large, larger than approximately 90 percent of meningiomas [3, 4, 9]. Next was the remarkable rate at which this meningioma grew. In a two-year span it increased in size from a negligible volume (less than 1 cm^3^) to a size to approximately 75 cm^3^ corresponding to a linear growth rate of 3 cm/year. In comparison, more than two-thirds of meningiomas grow less than 1 cm^3^ a year, and there have been very few other case reports in the literature with a linear growth rate of more than 1 cm/year [3]. Clomiphene upregulates the GnRH-FSH/LSH axis, which would increase circulating levels of estradiol and progesterone [10]. Furthermore, the patient was also taking exogenous progesterone as part of the fertility treatment as well. It can be inferred that both of these drugs could potentiate the growth of the meningioma and explain the meningioma's remarkable speed of growth.

There have been mixed results whether exogenous sex steroids affect the incidence of meningiomas [11–13]. There has been little research, however, into the relationship between sex steroids and the rate of meningioma growth. However, based on cases like this one and others that are similar, there is reason to believe that sex steroids can radically potentiate the growth of previously existing meningiomas.

Further investigation is needed to elucidate the complex relationship between fertility treatments and the incidence and progression of meningiomas. When women with meningiomas receive fertility treatment, it is very likely that the meningiomas will grow at a fast rate and will make it extremely difficult to be treated surgically. It will also expose patients to high risks of neurological deficit from both rapid tumor growth and from surgical treatment of such giant meningiomas. Patients with a prior or family history of meningioma should use hormonal therapy for treatment of infertility with caution, and we suggest in this specific case that they should be screened if they decide to proceed with the treatment. Furthermore, there is a potential risk for all women that undergo fertility treatment. This risk has not been studied enough.

## 4. Conclusion

Although further study is needed of this small subgroup of patients (women receiving fertility treatment), we recommend a screening imaging of brain with MRI be considered in the middle or toward the end of fertility treatments, especially if patients have had a prior or family history of meningioma. Early referral to neurosurgery and close observation of these meningiomas is essential as they can grow to an enormous size.
